# 31.2 CAN WE IMPROVE FUNCTIONAL OUTCOME AND ADHERENCE IN OPTIMISE PARTICIPANTS WITH A PSYCHOSOCIAL INTERVENTION?

**DOI:** 10.1093/schbul/sby014.128

**Published:** 2018-04-01

**Authors:** Richard Drake, Merete Nordentoft, Gill Haddock, John Ainsworth, Shon Lewis

**Affiliations:** 1 MAHSC; 2 Mental Health Centre Copenhagen; 3 University of Manchester

## Abstract

**Background:**

We hypothesised that a multi-modal psychosocial intervention (PSI) after first episodes of non-affective psychosis would increase antipsychotic adherence, improve functioning and prevent readmission in a multi-centre, blind-rated, randomised controlled trial.

**Methods:**

Following treatment of first episode non-affective psychosis with amisulpride, olanzapine or clozapine (those remitting after phases 1–3 of the OPTIMISE program or dropping-out but willing to enter the adherence trial) patients with DSM-IV schizophreniform disorder, schizophrenia, or schizoaffective disorder were eligible for allocation to PSI or treatment as usual (TAU).

PSI involved: i) e-learning via a psychoeducational website; ii) mHealth intervention with 3 months SMS medication reminders configured by participants; iii) motivational interviewing targeting adherence over 6 weeks.

Primary outcome measures at 3 and 12 month follow-up were Compliance Rating Scale (score 1–7 worst to best), and Social and Occupational Function Assessment Scale (SOFAS; 0–100). Secondary outcomes included remission, Drug Attitudes Inventory (DAI), and EuroQoL quality of life scale. We present interim analyses of 3 month data with general linear and logistic regression models, clustered by centre and adjusted for baseline scores and demographics.

**Results:**

Recruitment time, now finished, was extended to reach target sample size, so 12 month data are incomplete. 258 were allocated to PSI or TAU. 18 dropped-out before baseline assessment: 240 entered the modified intention to treat analysis (PSI 121, TAU 119). After PSI, 71% were followed-up at 3 months; after TAU, 80%. No baseline variable significantly predicted this attrition.

Webpages covering illness and treatment had 244–290 hits each. Only 24% of the PSI group set up SMS text reminders. At least 70% attended motivational interviewing, 77% of these for all sessions. Mean California Patient Alliance Scale item score was 5.2 (95% Confidence Interval, CI, 5.0, 5.3; score 1–7 poor-good).

Mean baseline SOFAS (SD) for the PSI group was 62 (14); for TAU 62 (15). General linear modelling of 12 week data included baseline CRS and drug attitudes: marginal mean SOFAS after PSI was 65.3 (CI 62.6, 68.0) and after TAU 61.4 (CI 59.3, 63.4; p0.025; standardised effect size Glass’ Δ=0.41). Median (IQR) compliance score was 6 (5,7) at baseline for PSI and 6 (6,7) for TAU; and 6 (5,7) at 3 months in both groups (ordinal logistic regression, p0.36).

In secondary analyses 3 month DAI did not differ significantly between PSI and TAU (marginal means 13.5 v 11.5, bootstrapped p0.164). EuroQoL wellbeing score was significantly better after PSI (marginal means 76.0 v 69.1, bootstrapped p0.003) and remission was significantly commoner (72 v 54%, binary logistic regression p0.007). No analysis result was sensitive to probability weighted adjustment for drop out.

**Discussion:**

Interim analyses indicate that immediately after PSI social function and wellbeing improved significantly and remission was commoner. Adherence and DAI did not differ significantly. Either PSI’s immediate benefits were non-specific or adherence measures failed to capture its effect. Longer term effects are unclear until definitive analyses planned before 2018.

